# Ischemia-Reperfusion under Hyperthermia Increases Heme Oxygenase-1 in Pyramidal Neurons and Astrocytes with Accelerating Neuronal Loss in Gerbil Hippocampus

**DOI:** 10.3390/ijms22083963

**Published:** 2021-04-12

**Authors:** Tae-Kyeong Lee, Jae-Chul Lee, Dae Won Kim, Bora Kim, Hyejin Sim, Jong Dai Kim, Ji Hyeon Ahn, Joon Ha Park, Choong-Hyun Lee, Moo-Ho Won, Soo Young Choi

**Affiliations:** 1 Department of Biomedical Science and Research Institute for Bioscience and Biotechnology, Hallym University, Chuncheon 24252, Gangwon, Korea; tk-lee@hallym.ac.kr; 2 Department of Neurobiology, School of Medicine, Kangwon National University, Chuncheon 24341, Gangwon, Korea; anajclee@kangwon.ac.kr (J.-C.L.); nbrkim17@gmail.com (B.K.); janny20@naver.com (H.S.); jh-ahn@ysu.ac.kr (J.H.A.); 3 Department of Biochemistry and Molecular Biology, Research Institute of Oral Sciences, College of Dentistry, Gangnung-Wonju National University, Gangneung 25457, Gangwon, Korea; kimdw@gwnu.ac.kr; 4 Division of Food Biotechnology, School of Biotechnology, Kangwon National University, Chuncheon 24341, Gangwon, Korea; jongdai@kangwon.ac.kr; 5 Department of Physical Therapy, College of Health Science, Youngsan University, Yangsan 50510, Gyeongnam, Korea; 6 Department of Anatomy, College of Korean Medicine, Dongguk University, Gyeongju 38066, Gyeongbuk, Korea; jh-park@dongguk.ac.kr; 7 Department of Pharmacy, College of Pharmacy, Dankook University, Cheonan 31116, Chungnam, Korea; anaphy@dankook.ac.kr

**Keywords:** glia, hippocampal subregions, hyperthermia, transient forebrain ischemia, heme oxygenase-1, neuronal death

## Abstract

It has been studied that the damage or death of neurons in the hippocampus is different according to hippocampal subregions, cornu ammonis 1–3 (CA1–3), after transient ischemia in the forebrain, showing that pyramidal neurons located in the subfield CA1 (CA1) are most vulnerable to this ischemia. Hyperthermia is a proven risk factor for brain ischemia and can develop more severe and extensive brain damage related with mortality rate. It is well known that heme oxygenase-1 (HO-1) activity and expression is increased by various stimuli in the brain, including hyperthermia. HO-1 can be either protective or deleterious in the central nervous system, and its roles depend on the expression levels of enzymes. In this study, we investigated the effects of hyperthermia during ischemia on HO-1 expression and neuronal damage/death in the hippocampus to examine the relationship between HO-1 and neuronal damage/death following 5-min transient ischemia in the forebrain using gerbils. Gerbils were assigned to four groups: (1) sham-operated gerbils with normothermia (Normo + sham group); (2) ischemia-operated gerbils with normothermia (Normo + ischemia group); (3) sham-operated gerbils with hyperthermia (39.5 ± 0.2 °C) during ischemia (Hyper + sham group); and (4) ischemia-operated gerbils with hyperthermia during ischemia (Hyper + ischemia group). HO-1 expression levels in CA1–3 of the Hyper + ischemia group were significantly higher than those in the Normo + ischemia group. HO-1 immunoreactivity in the Hyper + ischemia group was significantly increased in pyramidal neurons and astrocytes with time after ischemia, and the immunoreactivity was significantly higher than that in the Normo + ischemia group. In the Normo + Ischemia group, neuronal death was shown in pyramidal neurons located only in CA1 at 5 days after ischemia. However, in the Hyper + ischemia group, pyramidal neuronal death occurred in CA1–3 at 2 days after ischemia. Taken together, our findings showed that brain ischemic insult during hyperthermic condition brings up earlier and severer neuronal damage/death in the hippocampus, showing that HO-1 expression in neurons and astrocytes is different according to brain subregions and temperature condition. Based on these findings, we suggest that hyperthermia in patients with ischemic stroke must be taken into the consideration in the therapy.

## 1. Introduction

The brain has neurons that are very vulnerable to transient ischemia (ischemia-reperfusion), which deprives the neurons of oxygen supply, decreases metabolic rate and gives an insufficient reserve of high-energy carbohydrates when compared to other tissues [1]. The hippocampus is one of the brain structures that is vulnerable to transient ischemia, with results showing that vulnerability differs for each hippocampal subfield (CA1–3); specifically, CA1 is the most vulnerable to transient ischemia, while CA2/3 are resistant to the ischemia [2,3,4]. In CA1, pyramidal cells (neurons) die 4–5 days after transient forebrain ischemia for 5 min in gerbils, and this unique process of the death of the pyramidal cells is termed “delayed neuronal death (DND)” [2,5]. The DND is due to diverse changes in the cells, such as DNA damage, inflammation, excitotoxicity and oxidative stress, following ischemia-reperfusion [6,7]. Nevertheless, the exact mechanisms of DND remain unclear.

Elevated body temperature (hyperthermia) is a major factor in enhancing neuronal damage or death (loss) after brain ischemic insults [8,9]. It has been suggested that hyperthermia leads to more severe brain injury following ischemia through calcium influx into neurons, releases of neurotoxic excitatory neurotransmitters and reactive oxygen species, and dysfunction of vascular permeability [8,9]. In ischemic stroke patients, it has been confirmed that hyperthermia enlarges brain infarction following ischemic stroke and worsens the outcomes of ischemic damage [10]. Preclinical data have suggested that post-ischemic hyperthermia leads to more harmful effects in animal models of ischemic insults including transient global brain ischemia [11], permanent focal brain ischemia [12] and transient focal brain ischemia [13]. In a gerbil model of transient ischemia in the forebrain (telencephalon), hyperthermia conditioning before and during the transient ischemia led to more severe neuronal damage and glial activation in the hippocampus [14]. For the mechanisms of ischemic damage under hyperthermia, it has been proposed that vascular permeability dysfunction, the releases of neurotoxic excitatory neurotransmitters, the production of reactive oxygen species, and calcium influx into neurons can lead to more severe tissue injury [15,16,17]. However, the exact mechanisms of brain tissues or cells following ischemia-reperfusion under hyperthermia are not yet fully understood.

Heme oxygenase (HO) is an essential enzyme for all eukaryotic organisms that depend on aerobic oxidation and electron transport via heme-containing proteins [18,19]. The HO system is a rate-limiting step in the change of the heme into biliverdin, carbon monoxide and free iron (Fe^2+^) [20]. HO-1 is a inducible isoform and is greatly increased in response to various cellular stresses, such as ischemia, hypoxia, inflammation and radiation [21]. It is well known that HO-1 activity and expression are increased by various conditioning stimuli in the brain [22,23]. HO-1 can play either a protective or deleterious role in the central nervous system (CNS), and its roles depend on the expression levels of enzymes [24].

However, few studies about the relationship between neuronal damage/death and HO-1 in the ischemic hippocampus induced during hyperthermia have been reported, although post-ischemic hyperthermia displays more harmful effects in animal models of ischemic brain insults. In this regard, the aim of this research was to examine the effects of hyperthermia during ischemia on HO-1 expression in the hippocampus following transient forebrain ischemia for 5 min under hyperthermia to examine whether HO-1 expression is related to more severe neuronal loss in the ischemic hippocampus when induced under hyperthermia using Western analysis and immunohistochemistry in gerbils, which were used as a model of transient forebrain ischemia [14,25].

## 2. Results

### 2.1. Higher HO-1 Levels in CA1–3 of Normo + Ischemia Group than Those in Hyper + Ischemia Group

#### 2.1.1. HO-1 Levels in CA1

**Figure 1:**
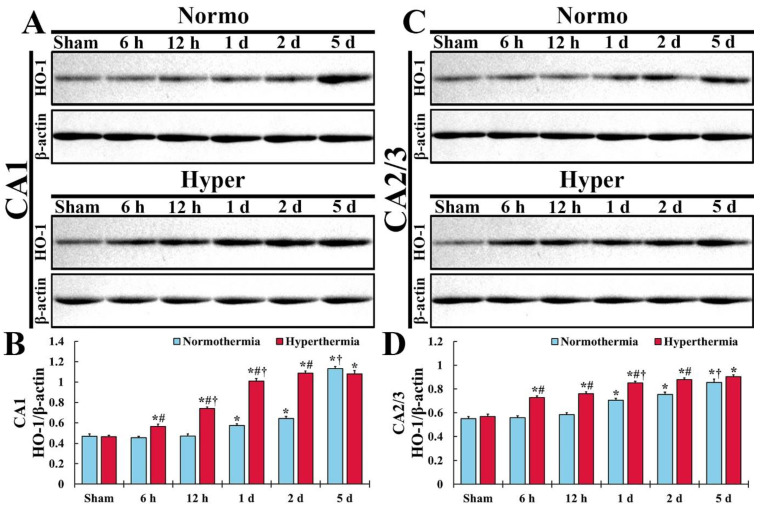
(**A**,**C**) Western blot analysis for HO-1 in CA1 (**A**) and CA2/3 (**C**) of the Normo + sham and Normo + ischemia, Hyper + sham and Hyper + ischemia groups at sham, 6 h, 12 h, 1 day, 2 days and 5 days after ischemia. In the Normo + ischemia group, HO-1 level is significantly increased from 1 day after ischemia in both CA1 and CA2/3. In the Hyper + ischemia group, HO-1 level is significantly increased from 6 h and higher than that in the Normo + ischemia group after ischemia, showing that the degree of the increased level is more apparent in CA1 than in CA2/3. (**B**,**D**) Normalized protein level versus β-actin in CA1 (**B**) and CA2/3 (**D**). Results are expressed as mean ± SEM. * *p* < 0.05 vs. Normo + sham group; ^#^ *p* < 0.05 vs. corresponding time point of Normo + ischemia group; ^†^ *p* < 0.05 vs. prior time point of each group (*n* = 5 at each time in each group).

In CA1 of the Normo + sham group, a fundamental expression level of HO-1 was detected, as shown in Figure 1A. In the Normo + ischemia group, HO-1 expression level was not altered until 12 h after ischemia (Figure 1A,B). In this group, a significant increase of HO-1 expression level (122.6% of the Normo + sham group) was shown at 1 day after ischemia compared with that in the Normo + sham group, and the expression level was not significantly changed at 2 days after ischemia (Figure 1A,B). Five days after ischemia, the expression level of HO-1 was dramatically elevated (242.1% of the Normo + sham group) compared with that in the Normo + sham group (Figure 1A,B).

In CA1 of the Hyper + sham group, the expression level of HO-1 was similar to that in the Normo + sham group (Figure 1A,B). In the Hyper + ischemia group, the expression level of HO-1 significantly and gradually increased until 2 days after ischemia, showing that the level at each point in time was higher (124.8% at 6 h, 157.4% at 12 h, 176.1% at 1 day, and 169.6% at 2 days) than that in the Normo + ischemia group (Figure 1A,B). However, at 5 days after ischemia, the expression level of HO-1 was similar to that in the Normo + ischemia group (Figure 1A,B).

#### 2.1.2. HO-1 Levels in CA2/3

In CA2/3 of the Normo + sham group, the baseline of HO-1 level was higher (117.8%) compared with that in CA1 of the Normo + sham group (Figure 1C,D). In the Normo + ischemia group, HO-1 expression level did not change until 12 h after ischemia compared with that in the Normo + sham group (Figure 1C,D). One day after ischemia, the expression level of HO-1 was significantly elevated (128.1%) compared with that in the Normo + sham group (Figure 1C,D). Thereafter, its expression level gradually increased (137.1% at 2 days, and 155.3% at 5 days) compared with that in the Normo + sham group (Figure 1C,D).

In CA2/3 of the Hyper + sham group, the expression level of HO-1 was also similar to that in the Normo + sham group (Figure 1C,D). In the Hyper + ischemia group, the expression level of HO-1 was significantly elevated from 6 h after ischemia, and higher (130.4% at 6 h, 130.0% at 12 h, 120.5%1 at 1 day, 116.4% at 2 days, and 105.7% at 5 days) than that in the Normo + ischemia group (Figure 1C,D).

### 2.2. Higher HO-1 Immunoreactivity in CA1–3 of Normo + Ischemia Group than That in Hyper + Ischemia Group

#### 2.2.1. HO-1 Immunoreactivity in CA1

**Figure 2:**
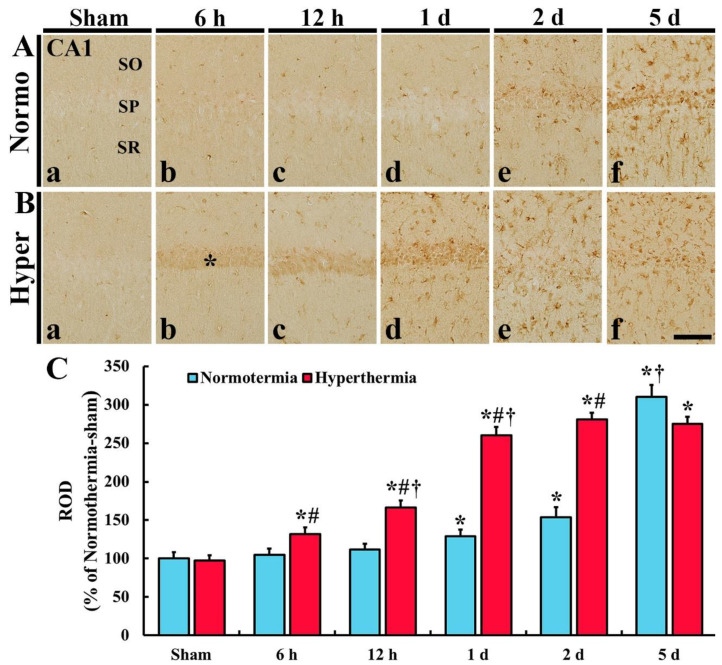
(**A**,**B**) Immunohistochemistry for HO-1 in CA1 of the Normo + sham, Normo + ischemia, Hyper + sham and Hyper + ischemia groups at sham (**Aa**,**Ba**), 6 h (**Ab**,**Bb**), 12 h (**Ac**,**Bc**), 1 day (**Ad**,**Bd**), 2 days (**Ae**,**Be**) and 5 days (**Af**,**Bf**) after ischemia. In the sham groups, HO-1 immunoreactivity is hardly shown. In the Normo + ischemia group, HO-1 immunoreactivity is significantly increased in all layers at 2 days and 5 days after ischemia. However, in the Hyper + ischemia group, HO-1 immunoreactivity increased in SP (asterisk) at 6 h to 1 day after ischemia, and in all layers from 1 day after ischemia. SO, stratum oriens; SP, stratum pyramidale; SR, stratum radiatum. Scale bar = 50 µm. (**C**) Relative optical density (ROD) of HO-1 immunoreactivity. Results are expressed as mean ± SEM. * *p* < 0.05 vs. Normo + sham group; ^#^ *p* < 0.05 vs. corresponding time point of Normo + ischemia group; ^†^ *p* < 0.05 vs. prior time point of each group (*n* = 5 at each time in each group).

In CA1, HO-1 immunoreactivity was hardly found in any layers of CA1 in the Normo + sham group (Figure 2Aa). In the Normo + ischemia group, HO-1 immunoreactivity was not altered until 12 h after ischemia (Figure 2Ab,c,C) and increased in cells throughout all layers of CA1 from 1 day after ischemia (Figure 2Ad–f), showing that the relative optical density (ROD) of HO-1 immunoreactivity was 154.0% at 2 days and 310.1% at 5 days after ischemia compared with that in the Normo + sham group (Figure 2C). In particular, HO-1 immunoreactive cells at 1 day, 2 days and 5 days seemed to be glial cells.

In the Hyper + sham group, there was no significant difference in HO-1 immunoreactivity in CA1 compared with that in the Normo + sham group (Figure 2Ba,C). In the Hyper + ischemia group, HO-1 immunoreactivity was significantly increased from 6 h after ischemia, and the immunoreactivity at each point in time was higher than that in the Normo + ischemia group, showing that the ROD was 166.2% at 12 h, 260.4% at 1 day, 280.7% at 2 days, and 275.8% at 5 days after ischemia compared with that in Normo + ischemia group (Figure 2Bb–f,C). In this group, HO-1 immunoreactivity was newly expressed in the cells located in the stratum pyramidale (SP) 6 h to 1 day after ischemia and in glial-like cells distributed in the other layers (strata oriens and radiatum, SO and SR) from 1 day after ischemia.

#### 2.2.2. HO-1 Immunoreactivity in CA2/3

**Figure 3:**
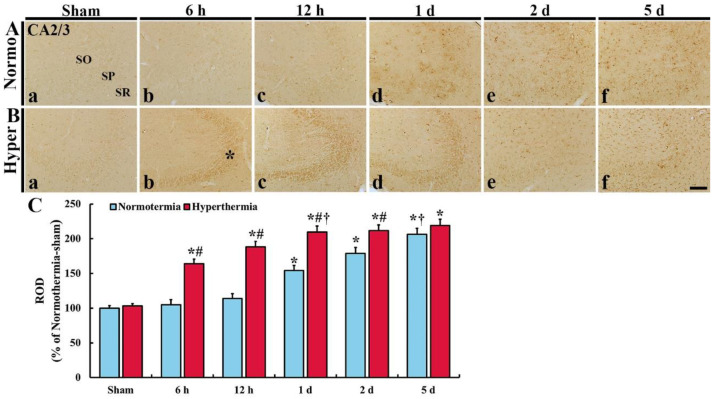
(**A**,**B**) Immunohistochemistry forHO-1 in CA2/3 of the Normo + sham, Normo + ischemia, Hyper + sham and Hyper + ischemia groups at sham (**Aa**,**Ba**), 6 h (**Ab**,**Bb**), 12 h (**Ac**,**Bc**), 1 day (**Ad**,**Bd**), 2 days (**Ae**,**Be**) and 5 days (**Af**,**Bf**) after ischemia. In the sham groups, HO-1 immunoreactivity is hardly shown. In the Normo + ischemia group, HO-1 immunoreactivity is apparently shown in all layers from 1 day after ischemia. However, in the Hyper + ischemia group, HO-1 immunoreactivity is apparently shown in SP (asterisk) at 6 h to 1 day, and in all layers from 2 days and 5 days after ischemia. SO, stratum oriens; SP, stratum pyramidale; SR, stratum radiatum. Scale bar = 50 µm. (**C**) Relative optical density (ROD) of HO-1 immunoreactivity. Results are expressed as mean ± SEM. * *p* < 0.05 vs. Normo + sham group; ^#^ *p* < 0.05 vs. corresponding time point of Normo + ischemia group; ^†^ *p* < 0.05 vs. prior time point of each group (*n* = 5 at each time in each group).

In CA2/3 of the Normo + sham group, HO-1 immunoreactivity was hardly found (Figure 3Aa). In the Normo + ischemia group, HO-1 immunoreactivity was not significantly altered until 12 h after ischemia compared with that in the Normo + sham group (Figure 3Ab,c,C). One day after ischemia, HO-1 immunoreactivity was apparently expressed in glia-like cells located in all layers, and the immunoreactivity gradually increased with time after ischemia, showing that the ROD was 154.3% at 1 day, 179.0% at 2 days and 206.2% at 5 days after ischemia compared with that in the Normo + sham group (Figure 3Ad–f,C).

In CA2/3 of the Hyper + sham group, HO-1 immunoreactivity was similar to that in the Normo + sham group (Figure 3Ba). In the Hyper + ischemia group, strong HO-1 immunoreactivity was shown in SP at 6 h, 12 h and 1 day after ischemia, and in all layers at 2 days and 5 days after ischemia, showing that the ROD was 164.0% at 6 h, 188.3% at 12 h, 209.8% at 1 day, 211.6% at 2 days, and 219.1% at 5 days after ischemia compared with that in the Normo + ischemia group (Figure 3Bb–f,C).

Interestingly, we found in this study that, although HO-1 immunoreactivity in CA1 and CA2/3 was gradually increased in both Normo+ and Hyper + ischemia groups, but the degree of the ROS in CA1 was significantly higher than that in CA2/3 (Figure 2C and Figure 3C).

### 2.3. Stronger HO-1 Expression in Pyramidal Neurons or Astrocytes of Hyper + Ischemia Group

**Figure 4:**
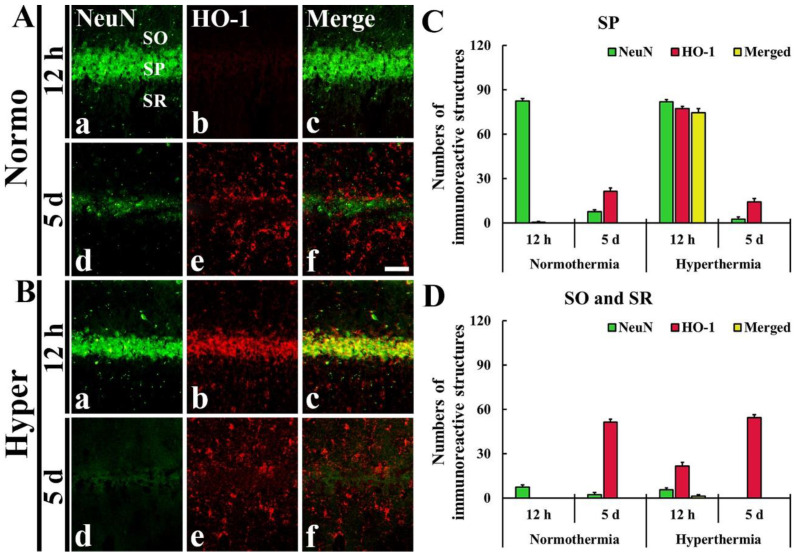
(**A**,**B**) Double immunofluorescence staining for NeuN (green, **Aa**,**Ad**,**Ba**,**Bd**) and HO-1 (red, **Ab**,**Ae**,**Bb**,**Be**), and merged image (**Ac**,**Af**,**Bc**,**Bf**) in CA1 at 12 h (**Aa**–**Ac**,**Ba**–**Bc**) and 5 days (**Ad**–**Af**,**Bd**–**Bf**) after ischemia. In the Hyper + ischemia group, HO-1 immunoreactivity was expressed in NeuN immunoreactive pyramidal neurons located in the stratum pyramidale (SP). SO, stratum oriens; SR, stratum radiatum. Scale bar = 50 µm. (**C**,**D**) The Mean numbers of double immunoreactive cells in the SP (**C**), and the SO and SR (**D**) (*n* = 5 at each time in each group).

**Figure 5:**
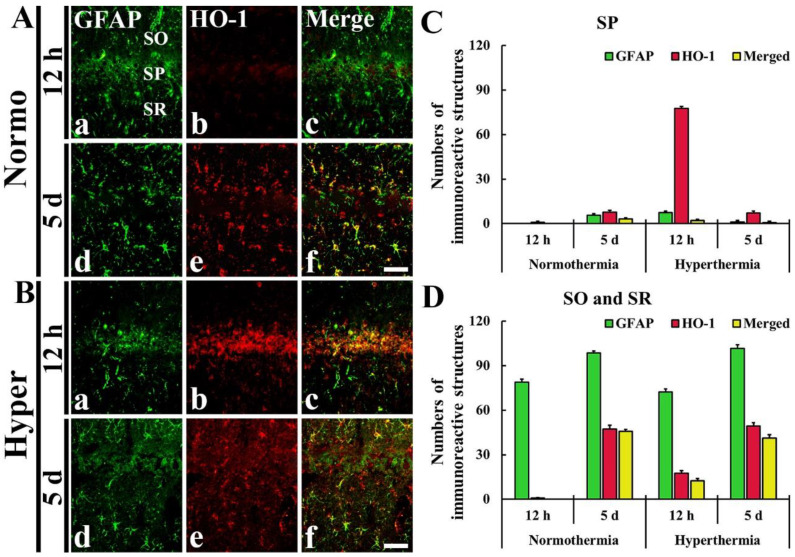
(**A**,**B**) Double immunofluorescence staining for GFAP (green, **Aa**,**Ad**,**Ba**,**Bd**) and HO-1 (red, **Ab**,**Ae**,**Bb**,**Be**), and merged image (**Ac**,**Af**,**Bc**,**Bf**) in CA1 at 12 h (**Aa**–**Ac**,**Ba**–**Bc**) and 5 days (**Ad**–**Af**,**Bd**–**Bf**) after ischemia. HO-1 immunoreactive cells are identified as GFAP immunoreactive astrocytes. SO, stratum oriens; SP, stratum pyramidale; SR, stratum radiatum. Scale bar = 50 µm. (**C**,**D**) The mean numbers of double immunoreactive cells in the SP (**C**), and the SO and SR (**D**) (*n* = 5 at each time in each group).

As described above, HO-1 immunoreactivity was newly expressed in the cells located in the SP, SO and SR after ischemia in both Normo + ischemia and Hyper + ischemia groups. To identify the cell types, we used double immunofluorescence staining with neuronal nuclei (NeuN, a marker for neurons) and glial fibrillary acidic protein (GFAP, a marker for astrocyte). The HO-1 immunoreactive cells located in the SP were identified as pyramidal neurons positive for NeuN (Figure 4), which are principal neurons in CA1–3, and the HO-1 immunoreactive cells located in the SO and SR were identified as astrocytes positive for GFAP (Figure 5).

#### 2.3.1. HO-1 Immunoreactivity Shown in Neuronal Nuclei (NeuN) Immunoreactive Pyramidal Neurons

In the Normo + ischemia group, NeuN immunoreactive neurons located in the SP were not positive for HO-1 at 12 h after ischemia (Figure 4Aa–f,C). However, in the Hyper + ischemia group, HO-1 immunoreactivity was shown in NeuN immunoreactive pyramidal neurons located in the SP at 12 h after ischemia, showing that most of the pyramidal neurons (about 75 cells/250 μm^2^) expressed HO-1 (Figure 4Ba–c,D).

#### 2.3.2. HO-1 Immunoreactivity Shown in Glial Fibrillary Acidic Protein (GFAP) Immunoreactive Astrocytes

In the Normo + ischemia group, GFAP immunoreactive astrocytes positive for HO-1 were rarely observed in the SP at 12 h after ischemia (Figure 5Aa–c,C). However, at 5 days after ischemia, many GFAP immunoreactive astrocytes (about 46 cells/250 μm^2^) expressed HO-1 in the SO and SR (Figure 5Ad–f,D).

In the Hyper + ischemia group, at 12 h and 5 days after ischemia, many GFAP immunoreactive astrocytes were positive for HO-1, showing that about 13 and 41 cells/250 μm^2^, respectively, were counted in the SO and SR (Figure 5Ba–f,D).

### 2.4. Deteriorated Loss of Pyramidal Neurons in CA1–3 of Hyper + Ischemia Groups

#### 2.4.1. Damaged Cresyl Violet-Positive (CV^+^) Cells in CA1–3

**Figure 6:**
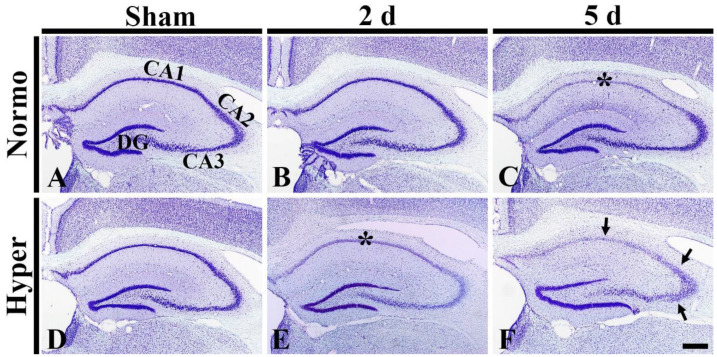
(**A**–**F**) CV staining in the hippocampus of the Normo + sham, Normo + ischemia, Hyper + sham and Hyper + ischemia groups at sham (**A**,**D**), 2 days (**B**,**E**) and 5 days (**C**,**F**) after ischemia. In the Normo + ischemia group, CV stainability is apparently pale (damaged) in pyramidal cells located in SP (asterisk) only in CA1 at 5 days after ischemia. In the Hyper + ischemia group, CV stainability is pale in SP (asterisk) in CA1–3 at 2 days after ischemia. Five days after ischemia, CV stainability in CA1–3 is more decreased in the stratum pyramidale (arrows). DG, dentate gyrus. Scale bar = 400 µm (*n* = 5 at each time in each group).

CV^+^ cells were well identified in the hippocampus, including CA1–3 in the Normo + sham group (Figure 6A). In the Normo + ischemia group, CV^+^ cells did not change in their distribution in CA1–3 until 2 days after ischemia (Figure 6B). Five days after ischemia, CV^+^ cells were apparently pale (damaged) in pyramidal cells (neurons) located in SP of CA1, but not in CA2/3 (Figure 6C).

In the Hyper + sham group, CV^+^ cells in the hippocampus were not different in their distribution from those in the Normo + sham group (Figure 6D). In the Hyper + ischemia groups, CV stainability in pyramidal neurons of CA1–3 was pale (damaged) at 2 days after ischemia (Figure 6E). At 5 days after ischemia, their CV stainability was more significantly reduced in CA1–3 (Figure 6F).

#### 2.4.2. Decreased NeuN^+^ Neurons in CA1–3

##### NeuN^+^ Neurons in CA1

**Figure 7:**
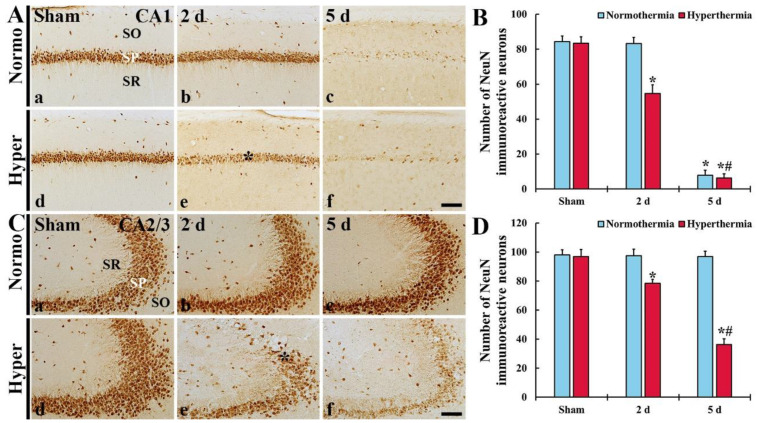
(**A**,**C**) NeuN immunohistochemistry in CA1 (**A**) and CA2/3 (**C**) of the Normo + sham, Normo + ischemia, Hyper + sham and Hyper + ischemia groups at sham (**Aa**,**Ad**,**Ca**,**Cd**), 2 (**Ab**,**Ae**,**Cb**,**Ce**) and 5 days (**Ac**,**Af**,**Cc**,**Cf**) after ischemia. In the Normo + ischemia group, NeuN^+^ pyramidal neurons are rarely found in SP only in CA1 at 5 days after ischemia. In the Hyper + ischemia group, NeuN^+^ neurons are decreased in numbers in the stratum pyramidale (SP) (asterisks) of CA1–3 and their immunoreactivity is reduced at 2 days after ischemia. At 5 days after ischemia, their numbers and immunoreactivity are much more reduced. SO, stratum oriens; SR, stratum radiatum. Scale bar = 100 µm. (**B**,**D**) Mean numbers of NeuN^+^ cells in CA1 (**B**) and CA2/3 (**D**). Results are expressed as mean ± SEM. * *p* < 0.05 vs. Normo + sham group; ^#^ *p* < 0.05 vs. corresponding time point of Normo + ischemia group (*n* = 5 at each time in each group).

In CA1 of the Normo + sham group, pyramidal neurons located in SP displayed strong immunoreactivity of NeuN (Figure 7Aa). In the Normo + ischemia group, NeuN^+^ pyramidal neurons were not altered in their distribution and immunoreactivity at 2 days after ischemia (Figure 7Ab). At 5 days after ischemia, however, most of the NeuN^+^ neurons disappeared (i.e., were severely damaged or had died) (Figure 7Ac).

In CA1 of the Hyper + sham group, pyramidal neurons were not different from those in the Normo + sham group (Figure 7Ad). However, in the Hyper + ischemia group, pyramidal neurons showed weak NeuN immunoreactivity and decreased in numbers (64.8% of the Normo + sham) at 2 days after ischemia (Figure 7Ae), and, at 5 days after ischemia, only a few NeuN^+^ neurons were detected (Figure 7Af,B).

##### NeuN-Immunoreactive (NeuN^+^) Neurons in CA2/3

In CA2/3 of in the Normo + sham group, pyramidal neurons were well immuno-stained with NeuN (Figure 7Ca). In the Normo + ischemia group, NeuN^+^ neurons in SP were not significantly changed compared with those found in the Normo + sham group until 5 days after ischemia (Figure 7Cb,c,D).

In CA2/3 of the Hyper + sham group, NeuN^+^ pyramidal neurons were not different from those in the Normo + sham group (Figure 7Cd). In the Hyper + ischemia group, NeuN^+^ neurons showed weak immunoreactivity and decreased in numbers (80.9% of the sham) compared with those in the Hyper + sham group (Figure 7Ce,2D). At 5 days after ischemia, NeuN^+^ neurons were more significantly decreased in numbers (37.4% of the sham) compared with those in the Hyper + sham group (Figure 7Cf,D).

#### 2.4.3. Increased Fluoro-Jade B-Positive (F-J B^+^) Cells in CA1–3

##### F-J B^+^ Cells in CA1

**Figure 8:**
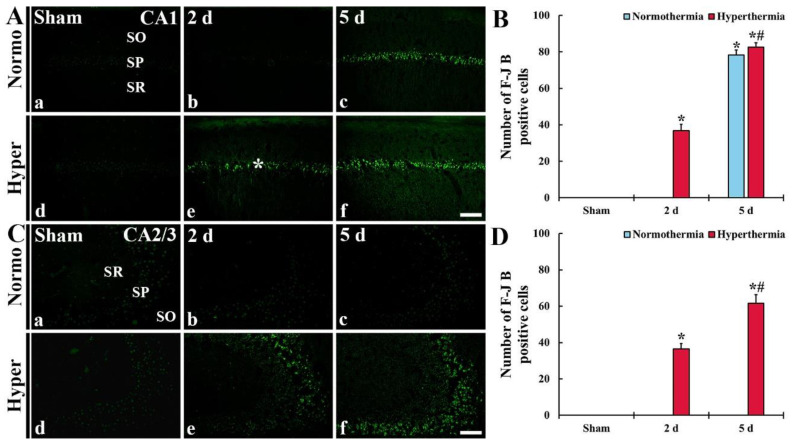
(**A**,**C**) Histofluorescence staining for F-J B in CA1 (**A**) and CA2/3 (**C**) of the Normo + sham, Normo + ischemia, Hyper + sham and Hyper + ischemia groups at sham (**Aa**,**Ad**,**Ca**,**Cd**), 2 days (**Ab**,**Ae**,**Cb**,**Ce**) and 5 days (**Ac**,**Af**,**Cc**,**Cf**) after ischemia. In the Normo + ischemia group, numerous F-J B^+^ cells are detected in SP only in CA1 at 5 days after ischemia. In the Hyper + ischemia group, many F-J B^+^ cells are shown in the stratum pyramidale (SP) of CA1–3 at 2 days after ischemia, and their numbers are markedly increased at 5 days after ischemia. SO, stratum oriens; SR, stratum radiatum. Scale bar = 100 µm. (**B**,**D**) Mean numbers of F-J B^+^ cells in CA1 (**B**) and CA2/3 (**D**). Results are expressed as mean ± SEM. * *p* < 0.05 vs. Normo + sham group; ^#^ *p* < 0.05 vs. corresponding time point of Normo + ischemia group (*n* = 5 at each time in each group).

In CA1 of the Normo + sham group, no F-J B^+^ cells (dead cells) were found in any layers (Figure 8Aa). In the Normo + ischemia group, F-J B^+^ cells were also not found at 2 days after ischemia (Figure 8Ab). At 5 days after ischemia, numerous F-J B^+^ cells were detected in SP (Figure 8Ac,B).

In CA1 of the Hyper + sham group, too, F-J B^+^ cells were not shown (Figure 8Ad). In the Hyper + ischemia group, many F-J B^+^ cells were detected in SP at 2 days after ischemia (Figure 8Ae). At 5 days after ischemia, the numbers of F-J B^+^ cells were significantly increased (224.0%) compared with those found at 2 days after ischemia (Figure 8Af,B).

##### F-J B^+^ Cells in CA2/3

In CA2/3 of the Normo + sham group, no F-J B^+^ cells were found (8Ca). In the Normo + ischemia group, also, F-J B^+^ cells were not found until 5 days after ischemia (8Cb and 8Cd).

In CA2/3 of, F-J B^+^ cells were not found (8Cd). However, many F-J B^+^ cells were detected in SP of the Hyper + ischemia group at 2 days after ischemia (Figure 8Ce,D). At 5 days after ischemia, their number was more increased (168.4% compared with that at 2 days after ischemia) (Figure 8Cf,D).

## 3. Discussion

In rodents, neurons in the hippocampal formation following transient global brain or forebrain ischemia are distinctly different in terms of their susceptibility to ischemic insult according to their hippocampal sub-regions [3]. Transient forebrain ischemia in gerbils is easily induced by temporary occlusion (5–15 min) of both common carotid arteries alone, because the gerbils lack the circle of the Willis formation between the carotid and vertebral circulations [2]. The pyramidal neurons, as principal neurons, in CA1 are most vulnerable to transient ischemia (ischemia and reperfusion, IR) injury; however, the pyramidal neurons in CA2/3 are essentially resistant to IR injury [2,3]. In our current study, we obtained the same findings of the loss of the pyramidal neurons in CA1 after 5-min transient forebrain ischemia under normothermia.

Hyperthermia is one of critical factors enhancing brain damage after brain ischemia [26,27]. Clinical studies have shown that ischemic patients with hyperthermia show expanded infarction in their brains and deteriorated outcomes of ischemic stroke [10,28]. There is multiple evidence showing that post-ischemic hyperthermia leads to more harmful effects in animal models of ischemic insults, including transient global brain ischemia [11], permanent focal brain ischemia [12], and transient focal brain ischemia [13]. Regarding the effects of hyperthermia during ischemia in animal models, Dietrich et al. (1990) and Kil et al. (1996) reported that hyperthermia during ischemia exacerbated the neuronal death of pyramidal neurons in CA1 after a rat model of transient global brain ischemia induced by four-vessel occlusion [11,29], and Meden et al. (1994) showed that hyperthermia during ischemia in a rat model of transient focal brain ischemia induced by occlusion of the internal carotid artery was more deleterious in the infarct [30]. In addition, we recently reported that elevating the body temperature for 30 min before/during transient forebrain ischemia exacerbated neuronal damage/death in the polymorphic layer of gerbil dentate gyrus compared with the neuronal damage under normothermic condition [14]. As described above, many studies on the effects of post-ischemic hyperthermia on ischemic outcomes have been carried out, but studies on the mechanisms of the effects of hyperthermia during ischemia on ischemic outcomes are insufficient.

HO-1 belongs to the response protein superfamily that catalyzes the rate-limiting step of heme degradation and produces iron and biliverdin in the brain and other tissues [31]. Previous studies have shown that the upregulation of HO-1 expression improves reperfusion injury in major organs (the heart, brain, liver, kidney, etc.) but HO-1 deficiency in patients induces the enhancement of systemic inflammation response [32,33]. In hearts, it has been reported that the up-regulation of HO-1 in a discordant cardiac xenograft inhibited apoptosis and alleviated inflammation [34]. In livers, Kato et al. (2001) reported that HO-1 upregulation in rats provided protection against liver damage induced by ischemia-reperfusion [35]. In brains, it has been reported that the overexpression of HO-1 in HO-1 transgenic rats attenuated ischemic stroke damage induced by middle cerebral artery occlusion [24]. In addition, Chen et al. (2000) reported that, in cerebellar granular neurons isolated from homozygous transgenic mice, increased HO-1 expression protected neuronal cells from oxidative stress [36]. These beneficial effects suggest that HO-1 is related to decreased free heme concentration and production of anti-oxidant compounds (biliverdin/bilirubin) [37,38].

However, in the central nervous system, HO-1 can be deleterious, depending on its expression level [24]. In normal brains, HO-2 expression is abundant, constitutive and fairly ubiquitous, while HO-1 mRNA and protein are confined to minor populations of neurons and neuroglial cells [37]. Under abnormal circumstances, endogenous HO-1 is induced and exacerbates neural injury. The HO-1 gene is exquisitely sensitive to induction by diverse pro-oxidant and other stressors [37]. In Parkinson disease, HO-1 is markedly overexpressed in astrocytes in the substantia nigra [37]. In a review paper by Schipper et al. (2009), in Alzheimer disease, HO-1 is hyperactive in the stressed astrocytes and promotes mitochondrial sequestration of non-transferrin iron, which may thereby contribute to pathological iron deposition [39]. In this sense, it has been reported that the pharmacological inhibitions of HO-1 activity attenuated pyramidal neuronal injury induced by metalloporphyrins in animal hippocampal CA1 slices [40] and recovered brain edema and necrosis following focal brain hypoperfusion in rats [41]. In our current study, HO-1 immunoreactivity was shown early and significantly increased in NeuN immunoreactive pyramidal neurons and GFAP immunoreactive astrocytes in CA1–3 of the Hyper + ischemia group when compared with that in the Normo + ischemia group. Therefore, this finding suggests that increased HO-1 expression in the Hyper + ischemia group accelerates neuronal damage/death in CA1–3 following ischemia-reperfusion induced under hyperthermia.

Thus, we compared neuronal damage/death in CA1–3 between the Normo + ischemia and Hyper + ischemia groups by NeuN immunohistochemistry and F-J B histofluorescence staining. In particular, F-J B has a good affinity for entirely degenerating neurons, and it is a very useful marker in studying neuronal degeneration in brains after ischemic insults [42]. In this study, F-J B histofluorescence staining informed us that hyperthermia during ischemia in the Hyper + ischemia group accelerated and exacerbated death of CA1 pyramidal neurons after ischemia-reperfusion. In addition, the pyramidal neurons in CA2/3, which were not dead in the Normo + ischemia group, apparently and markedly died from 2 days after ischemia-reperfusion. For neuronal death following hyperthermia after ischemia-reperfusion, it was reported that accelerated and exacerbated death of pyramidal neurons in CA1 was shown in gerbils in the case where the body temperature was raised (38.5–39.5 °C) during after ischemia-reperfusion [14,43]. Taken together, we suggest that hyperthermia during ischemia must increase the extent and severity of neuronal death in CA1–3.

In conclusion, hyperthermic condition (39.5 ± 0.2 °C) during transient ischemia in gerbil forebrain significantly increased HO-1 expression in pyramidal neurons and astrocytes in the CA 1-3 compared with that under normothermic conditions (37 ± 0.2 °C). Simultaneously, a severe and extensive loss (death) of the pyramidal neurons following ischemia-reperfusion was found in the Hyper + ischemia group compared with that in the Normo + ischemia group. These findings indicate that increased HO-1 in hippocampal pyramidal neurons and astrocytes in ischemic regions after ischemia-reperfusion under hyperthermia must be deleterious, not protective. Therefore, we suggest that, in patients with ischemic stroke, hyperthermic fact during ischemia must be considered for the therapy.

## 4. Materials and Methods

### 4.1. Experimental Animals

Male gerbils at 6.5 months of age (body weight, 78 ± 5 g) were provided by the “Experimental Animal Center (Kangwon National University, Chuncheon, Gangwon, South Korea). The gerbils were housed (*n* = 3–5 animals per cage) in standard conditions (room temperature, 23 ± 0.5 °C; humidity, 55 ± 5%; 12:12 light/dark cycle), and they had free access to pellet feed and water. Entrance to the room was strictly controlled in order to reduce inessential stress to the gerbils.

Experimental protocol with the gerbils was approved (approval number, KW-180124-1; approval date, 18 February 2020) by Institutional Animal Care and Use Committee of Kangwon National University. In addition, animal care and handling was performed in accordance with the guidelines of the “Current International Laws and Policies” in the “Guide for the Care and Use of Laboratory Animals” [44].

### 4.2. Experimental Groups

The gerbils (total *n* = 120) for the experiment were divided into four groups: (1) Normo + sham group (*n* = 10), which was given no ischemia (sham operation) at normothermia (37 ± 0.2 °C); (2) Normo + ischemia group (*n* = 50), which was given a 5-min transient forebrain ischemia at normothermia; (3) Hyper + sham group (*n* = 10), which was given sham operation at hyperthermia (39.5 ± 0.2 °C); (4) Hyper + ischemia group (*n* = 50), which was given ischemia at hyperthermia.

Hyperthermic conditioning was induced by subjecting the gerbils to a heating pad, which was connected to a rectal thermistor (TR-100; Fine Science Tools, Foster City, CA, USA), until the temperature reached 39.5 ± 0.2 °C under anesthesia. The hyperthermia conditioning was maintained for 30 min before and during ischemia. The gerbils in each ischemia group were given recovery times of 6 h (*n* = 10/group), 12 h (*n* = 10/group), 1 day (*n* = 10/group), 2 days (*n* = 10/group) and 5 days (*n* = 10/group) after ischemia, because pyramidal cells in CA1 of the Normo + ischemia group die from 4 or 5 days after a 5-min transient forebrain ischemia [14]. The gerbils in each sham group were given recovery times of 0 h (*n* = 10) and 5 days (*n* = 10) after ischemia.

### 4.3. Induction of a 5-Min Transient Forebrain Ischemia

A 5-min transient ischemia was developed in the forebrain according to our published method [7]. In brief, the gerbils for the ischemia were anesthetized with a mixture of 2.5% isoflurane in 67% nitrous oxide and 33% oxygen, which does not affect body temperature. The ischemia was developed by simultaneously occluding both common carotid arteries using aneurysm clips (Yasargil FE 723K) (Aesculap, Tuttlingen, Germany). After occlusion for 5 min, the clips were removed from the arteries. To confirm perfect ischemia, the complete stop of blood flow was observed in the central artery in both retinae using ophthalmoscope (HEINE K180^®)^) from Heine Optotechnik (Herrsching, Germany). To control temperature, the gerbils of the Normo and Hyper groups were on a heating pad at 37 ± 0.2 °C and 39.5 ± 0.2 °C respectively, during the surgery. After the surgery, the gerbils were kept in incubators (Mirae Medical Industry, Seoul, South Korea) with 23 °C and 60% humidity until body temperature was normothermic. The gerbils of the sham groups were given the surgery without the occlusion of the arteries.

### 4.4. Western Blot Analysis

Western blots were carried out according to methods described in [45,46]. In short, five gerbils/group were deeply anesthetized by an intraperitoneal injection of 90 mg/kg pentobarbital sodium (JW pharm. Co., Ltd., Seoul, Korea) [47] at each point in time after ischemia. Under the anesthesia, their brains were removed, and the tissues of CA1 and CA2/3 were respectively dissected using surgical blades. The obtained tissues were homogenized with 0.05 M PBS (pH 7.4) containing 0.1 mM ethylene glycol-bis (β-aminoethyl ether)-N,N,N′,N′-tetraacetic acid (EGTA) (pH 8.0), 10 mM ethylenediaminetetraacetic acid (EDTA, pH 8.0), 0.2% Nonidet P-40, 100 mM β-glycerophosphate, 15 mM sodium pyrophosphate, 2 mM sodium orthovanadate, 50 mM NaF, 150 mM NaCl, 1 mM phenylmethylsulfonyl fluoride (PMSF), and 1 mM dithiothreitol (DTT). The homogenized CA1 and CA2/3 tissues were centrifuged, and their supernatants were taken for assessing protein level using a Micro BCA kit with bovine serum albumin (Pierce Chemical, Rockford, IL, USA) as the standard. The aliquots including total protein (20 μg) were boiled in loading buffer consisting of 150 mM Tris (pH 6.8), 0.3% bromophenol blue, 3 mM DTT, 6% SDS, and 30% glycerol. Thereafter, the samples were separated using 10% sodium dodecyl sulfate-polyacrylamide gel electrophoresis (SDS-PAGE), and the gels were transferred to nitrocellulose membranes from Pall Company (East Hills, NY, USA) at 350 mA under 4 °C for 1.5 h. In this experiment, to avoid non-specific staining, the nitrocellulose membranes were immersed into 5% defatted milk at 23 °C for 1 h. Next, those membranes were immunoreacted with each primary antibody at 4 °C for 8 h: (1) rabbit anti-HO-1 (diluted 1:2000; Abcam, Cambridge, UK) and (2) rabbit anti-β-actin (diluted 1:2000; Sigma-Aldrich, St. Louis, MO, USA). In succession, the membranes were incubated with HRP-conjugated donkey anti-rabbit IgG (diluted 1:5000; Santa Cruz Biotechnology, Santa Cruz, CA, USA) at room temperature for 1 h. Finally, a luminol-based chemiluminescence kit from Pierce (Thermo Fisher Scientific Inc., MA, USA) was used for enhancement of visualization.

### 4.5. Preparation of Histological Sections

For histological examination, five gerbils/group at each point in time after ischemia were deeply anesthetized with pentobarbital sodium as described above. Under the anesthesia, the gerbils were rinsed with 0.1 M phosphate-buffered saline (PBS, pH 7.4) via the left ventricle and fixed with 4% paraformaldehyde (in 0.1 M phosphate-buffer (PB), pH 7.4). Their brains were removed and more fixed in the same fixative for 4 h. To obtain coronal sections containing the hippocampus, the brains were trimmed, embedded in tissue-freezing medium and serially sectioned into 30-µm thickness in cryostat of Leica (Wetzlar, Germany).

For the histological analyses, sections with hippocampi were made between levels of −1.4 mm and −2.2 mm based on Bregma according to the “Gerbil Brain Atlas” by Radtke-Schuller (2016) [48].

### 4.6. Immunohistochemistry

Immunohistochemistry for our current study, the streptavidin-biotin-peroxidase method was used for the detection of NeuN (a marker for neurons) and HO-1 according to the method by [49] with modification. Representative brain sections were selected at the level of -1.8 and 2.7 mm antero-posterior to the bregma according to the gerbil brain atlas [48]. The sections were rinsed in 0.1 M PBS (pH, 7.4) three times. Endogenous peroxidase activity was blocked with 0.3% hydrogen peroxide for 25 min at room temperature. In addition, unspecific proteins were blocked using 5% normal horse serum for 30 min at room temperature. Incubation with primary antibodies mouse anti-NeuN (diluted 1:1000) (Chemicon, Temecula, CA, USA) and rabbit anti-HO-1 (diluted 1:100) (Abcam, Cambridge, MA, USA) was performed at 4 °C overnight. These sections were then washed in 0.1 M PBS three times. Incubation with secondary antibody (biotinylated horse anti-mouse IgG and/or horse anti-sheep IgG (diluted 1:250) (Vector, Burlingame, CA, USA) and streptavidin peroxidase complex (diluted 1:200) (Vector, Burlingame, CA, USA) was performed for 30 min at room temperature. Lastly, the immunoreacted tissues were visualized by solution of 3,3′-diaminobenzidine tetrahydrochloride (Sigma-Aldrich, St. Louis, MO, USA). These sections were rinsed, dehydrated in 70%, 80%, 90%, 95%, 100% ethyl alcohol, cleared in xylene and coverslipped with Permount (Thermo Fisher Scientific Inc., MA, USA) in a fume hood.

For the negative control, the same brain tissues were incubated with the pre-immune serum except for each primary antibody. When the tissues were examined, there were no immunostained structures in the tested tissues (data not shown).

### 4.7. Double Immunofluorescence Staining

To determine what types of cells contained HO-1, double immunofluorescence staining was carried out. Primary antibodies were rabbit GFAP (a marker for astrocyte) (diluted 1:800) (Abcam, Cambridge, UK), rabbit anti-NeuN (a marker for neuron) (diluted 1:1000) (Merck Millipore, Burlington, MA, USA) and mouse anti-HO-1 (diluted 1:100) (Abcam, Cambridge, MA, USA). As described above, endogenous peroxidase activity and unspecific proteins in the sections were blocked. These sections were immunoreacted with the mixture of Alexa Fluor^®^ 546-conjugated goat anti-rabbit IgG (diluted 1:500) (Invitrogen, Waltham, MA, USA) and Alexa Fluor^®^ 546-conjugated donkey anti-mouse IgG (diluted 1:500) (Invitrogen, Waltham, MA, USA). Immediately, these immunoreacted brain sections were mounted on the slide glasses and dehydrated with dry oven (WiseVen^®^ WOC High Clean Air Oven) (Daihan Scientific Co., Ltd., Gangwon, Korea). Finally, the sections were coverslipped with Canada balsam (Kanto Chemical Co. Inc., Tokyo, Japan).

### 4.8. CV Staining

CV is an organic compound with the chemical formula C_19_H_18_ClN_3_O. It is a basic dye and used as a common stain in histology. In this experiment, CV staining was used to investigate morphological changes in cells distributed in the hippocampus containing CA1–3.

CV staining was done to examine changes in cellular distribution and morphology in CA1–3 according to the method [49]. Briefly, the sections were stained in 0.1% (*w/v*) CV acetate (Sigma-Aldrich Co., St. Louis, MO, USA) for 15 min at room temperature and rinsed in distilled water. Decolorization was done in 70% ethyl alcohol for few seconds. Immediately, these sections were dehydrated in 80%, 90%, 95%, and 100% ethyl alcohol, cleared in xylene and mounted with Canada balsam (Sigma-Aldrich Co., St. Louis, MO, USA).

### 4.9. Histofluorescence Staining with F-J B

F-J B is a polyanionic fluorescein derivative that sensitively and specifically binds to degenerating neurons. It is a dark red power and has a green iridescence with excitation peak at 480 nm and emission peak at 525 nm. In this study, F-J B histofluorescence was performed to examine death (loss) of cells in CA1–3 following ischemia.

Histofluorescence staining with F-J B (a marker for degenerating or dead cells) was performed to examine death (loss) of cells in CA1–3 following ischemia according to the methods by Schmued and Hopkins [42] and Anderson, et al. [50] with a slight modification. In short, the brain sections were incubated in 0.06% potassium permanganate on a rotating stage for ten minutes, rinsed in distilled water for two minutes and stained with 0.0004% F-J B (Histochem, Jefferson, AR, USA) for twenty minutes. These were rinsed in distilled water and put down on slide warmers until they were fully dried. The tissues on the slides were cleared by immersion in xylene and coverslipped with DPX (Fluka, Milwaukee, WI, USA).

### 4.10. Data Analysis

All measurements in this experiment were performed to insure the objectivity in blind conditions by three observers under the same conditions.

Immunoblots of HO-1 were analyzed according to a method by [51]. In short, using Scion Image software (Scion Crop., Frederick, MD, USA), the bands were respectively scanned, and then densitometric analysis was performed. In this experiment, protein level was normalized versus the corresponding level of β-actin.

For the analysis of HO-1 immunohistochemistry, the density of HO-1 immunoreactive structures were evaluated in CA1 and CA2/3. Digital images of HO-1 immunoreactive structures were taken at the middle of CA1 and CA2/3 using AxioM1 light microscope (Carl Zeiss, Oberkochen, Germany), which had a digital camera connected to a PC monitor. The video images were digitized into an array of 512 × 512 pixels, and each pixel resolution was 256 gray levels (white to dark signal corresponded from 255 to 0). According to a method by [52], the density of the immunoreactive structures was evaluated as optical density (OD) (OD = log 256/mean gray level). The background density was subtracted, and the ratio of the OD was calibrated using Adobe Photoshop 8.0, analyzing as a percent (relative optical density, ROD), with the Normo + sham group (100%).

For the analysis of NeuN^+^ neurons, the images of NeuN^+^ cells were taken in CA1 and CA2/3 using AxioM1 light microscope as described above. The numbers of NeuN^+^ neurons were counted in 250 µm^2^ at the middle of the SP in CA1 and CA2/3. Cell count of the NeuN^+^ neurons was done by averaging their total numbers obtained from all observed sections. In addition, the numbers of NeuN/HO-1 immunoreactive cells (neurons expressing HO-1) and FGAP/HO-1 immunoreactive cells (astrocytes expressing OH-1), the images of the co-localized cells were counted in a representative area of 250 μm^2^ at each layer of CA1–3.

To analyze F-J B^+^ cells, the images of the F-J B^+^ cells were captured at the representative area in the SP in CA1 and CA2/3 using fluorescence microscope of Carl Zeiss (Göttingen, Germany) equipped with blue excitation fluorescence filter between 450–490 nm. The images of F-J B^+^ cells were counted with image analysis software (Rasband, W.S., ImageJ, United States National Institute of Health).

### 4.11. Statistical Analysis

In this study, the data were expressed as the mean ± SEM. The differences of the means among the experimental groups were statistically analyzed by analysis of variance (ANOVA) with a post hoc Bonferroni’s multiple comparison tests with SPSS program to elucidate ischemia-related differences among the groups. In addition, to compare two independent variables between Normo and Hyper groups, and their interaction, two-way ANOVA was used with the Bonferroni *post hoc*. *p* < 0.05 was used for statistical significance.

## Data Availability

The data presented in this study are available on request from the corresponding author.

## Abbreviations

CA, cornu ammonis; CNS, central nervous system; CV, cresyl violet; DND, delayed neuronal death; F-J B, fluoro-Jade B; GFAP, glial fibrillary acidic protein; HO-1, heme oxygenase-1; NeuN, neuronal nuclei; ROD, relative optical density; SO, stratum oriens; SP, stratum pyramidale; SR, stratum radiatum
